# Solid-phase microextraction as a green sample preparation strategy for volatile organic compound profiling in coffee: a review with AGREEprep-based evaluation

**DOI:** 10.1038/s41538-026-00984-4

**Published:** 2026-07-31

**Authors:** Yerkanat Syrgabek, Adrián Fuente-Ballesteros, Vânia G. Zuin Zeidler

**Affiliations:** 1 Center of Physical-Chemical Methods of Research and Analysis, Al-Farabi Kazakh National University, Almaty, Kazakhstan; 2 Department of Fundamental Medicine, Faculty of Medicine and Healthcare, Al-Farabi Kazakh National University, Almaty, Kazakhstan; 3 Institute of Sustainable Chemistry, School of Sustainability, Leuphana University Lüneburg, Lüneburg, Germany

**Keywords:** Chemistry, Engineering, Environmental sciences

## Abstract

Solid-phase microextraction (SPME) is a solvent-free sample preparation technique increasingly applied in coffee analysis. This review examines recent studies (2020–2025) on SPME for volatile organic compound profiling in coffee. Coupled with GC-MS and chemometrics, SPME supports aroma characterization, authenticity evaluation, and sample classification. AGREEprep scores (0.29–0.73) suggest moderate greenness, highlighting SPME as a promising strategy with potential for greener and more sustainable coffee analysis.

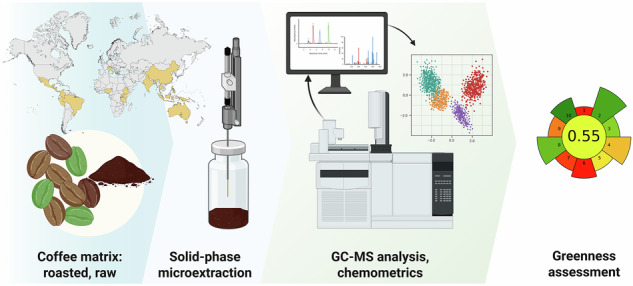

## Introduction

Global coffee consumption, estimated by the International Coffee Organization (ICO) at two billion cups per day, makes it the second most valuable traded commodity worldwide^1,2^. Today, coffee consumption transcends purely material aspects, as it can also be understood as a cultural and identity-defining element, which seeks to promote “life experiences”, particularly those that are considered rarer and more expensive. Viewed as both a cultural heritage and a commodity with strong global consumer appeal, coffee holds a value surpassed only by oil on the world market^3,4^. Coffee quality can be a multidimensional construct integrating aroma, flavor, and mouthfeel, formally defined within the Specialty Coffee Association (SCA) framework as coffee scoring more than 80 points in a standardized sensory evaluation^5–7^. This quality profile arises from interactions among genotype, terroir, and technological interventions throughout the production chain^1^. Establishing relationships between green bean chemical precursors and sensory outcomes is essential for authentication and industrial quality control^8,9^. Aroma constitutes the primary determinant of market value and derives from more than 1000 volatile organic compounds (VOCs) generated during processing and roasting, although only about 5% influence sensory perception^7,10^. Furans (sweet, caramel-like) and pyrazines (roasted, nutty) dominate key aroma notes. Non-volatile compounds determine taste and mouthfeel; caffeine and trigonelline contribute to bitterness, organic acids define acidity, and chlorogenic acids influence astringency^11^. Terroir variables, particularly altitude, temperature, and rainfall, modulate bean density and precursor composition, and high-altitude cultivation is generally associated with greater flavor complexity^1^. Post-harvest processing modifies sugar and amino acid availability through fermentation, whereas roasting induces Maillard reactions and Strecker degradation, often exerting a stronger influence on aroma than geographical origin itself^5,12^.

Conventional analysis of coffee VOCs is primarily based on liquid-liquid extraction (LLE) and solid-phase extraction (SPE), both associated with substantial organic solvent consumption, hazardous waste generation, and elevated operational costs^13,14^. These solvent-based approaches, together with purge-and-trap and stir bar sorptive extraction (SBSE), involve labor-intensive pretreatment procedures that reduce analytical throughput and increase the risk of analyte loss or contamination^13,15^. Thermal techniques, including steam and vacuum distillation, are limited by prolonged extraction times and the possible degradation of thermolabile compounds or the formation of heat-induced artifacts. Although static headspace represents a solvent-free alternative, its limited sensitivity prevents the detection of trace odorants with high odor activity values (OAVs) in complex roasted matrices. Furthermore, many conventional methods are destructive, which restricts the integration of chemical profiling with complementary physical or sensory analyses of the same sample. While solvent-assisted flavor evaporation (SAFE) achieves high recovery efficiency, its experimental complexity and requirement for specialized instrumentation limit its routine industrial application. Together with the time-intensive and subjective nature of conventional cupping protocols^16^, these technical, economic, and environmental constraints indicate the need for efficient, sustainable, and reproducible sampling platforms capable of generating reliable molecular fingerprints for multidimensional coffee quality assessment.

Beyond conventional extraction methods, a wide range of sample preparation and detection techniques has been used for coffee quality assessment. For volatile compounds, common approaches include LLE, dispersive liquid–liquid microextraction (DLLME), SPE, simultaneous distillation–extraction (SDE), and headspace sorptive extraction (HSSE). Each method differs in selectivity, sensitivity, and environmental impact^17^. For non-volatile compounds such as caffeine, chlorogenic acids, trigonelline, and organic acids, high-performance liquid chromatography (HPLC) with ultraviolet or diode array detection remains the standard method^17^. Spectroscopic techniques, including near-infrared (NIR), Fourier-transform infrared (FTIR), nuclear magnetic resonance (NMR), and Raman spectroscopy, are increasingly used as rapid and non-destructive tools for authentication, roast classification, and defect detection^18^. Sensor-based systems such as electronic noses and electronic tongues provide fast chemical fingerprints and support real-time evaluation of aroma and taste. Imaging methods, including hyperspectral and multispectral techniques, are also used for visual quality assessment of green and roasted coffee beans. Recent reviews have summarized both targeted and untargeted approaches for coffee analysis and highlighted the growing use of AI-based and portable technologies. However, these methods address different aspects of coffee quality. In contrast, solid-phase microextraction (SPME) offers solvent-free operation, simple sample preparation, and direct compatibility with GC-MS for volatile analysis, which makes it well suited for green analytical applications in coffee research^17,18^.

SPME represents a green, solvent-free sample preparation procedure for coffee VOCs, eliminating the hazardous solvent consumption associated with LLE while reducing waste generation and operator exposure^13,14,19^. Polymer-coated fused silica fibers can be reused for hundreds of thermal conditioning cycles, which supports environmental and economic sustainability^16^. Advanced coatings, particularly divinylbenzene/carboxen/polydimethylsiloxane (DVB/CAR/PDMS), enable broad-spectrum extraction of VOCs across a wide range of polarities and molecular weights, allowing efficient recovery of key aroma markers from complex coffee matrices^1,11,14,20^. The development of SPME Arrow technology, characterized by a five- to twenty-fold larger sorbent phase, further increases sensitivity for trace odorants^5,15,21^. When combined with gas chromatography–mass spectrometry (GC–MS) and chemometric modeling, SPME enables non-destructive molecular fingerprinting of green and roasted beans for origin authentication and quality classification^1,11,13,16,22^. This methodology links subjective sensory evaluation to reproducible chemical profiling. However, analytical robustness requires optimization of thermodynamic and kinetic parameters, including extraction temperature, equilibration time, and sample mass^19,20^. Moreover, SPME has become an established green technique for both research and industrial coffee quality control^22^.

This review aims to examine SPME as a green sample preparation strategy for coffee analysis and quality control, based on a critical literature review. It evaluates the role of SPME in volatile profiling for aroma characterization, origin authentication, roast discrimination, freshness assessment, and storage monitoring. Attention is paid to the environmental, analytical, and operational advantages of SPME compared with conventional solvent-based methodologies in the framework of green analytical chemistry principles and approaches^23^. In addition, the greenness of sample preparation strategies reported in 34 selected studies is evaluated using the AGREEprep metric, which serves green performance indicator (see Table 1).

**Table 1:** Comprehensive summary of SPME methods for coffee analysis with AGREEprep scores

#	No. of analytes	Target analyte class	Coffee type (origin)	Sample matrix	SPME fiber type, extraction conditions, instrumentation	Primary application	Key findings	AGREEprep pictograms	Ref.
1	25	Pyrazines, furanic compounds, organic acids, alcohols, and aldehydes	Date seed coffee alternative (Australia)	Roasted date seed powder and brewed beverage	PDMS/DVB; 60 °C equilibration for 15 min followed by 15 min extraction; HS–SPME–GC–MS	Roasting effects	Total volatile concentrations generally declined with increased roasting, although furfural remained a stable aroma contributor		^40^
2	2275	Lipids, organic acids, organoheterocyclics, terpenoids, heterocyclic compounds, esters, ketones	Arabica coffee (China)	Roasted coffee beans	DVB/CWR/PDMS; 60 °C extraction for 15 min; HS–SPME–GC–MS	Fermentation impact	Key odor-active contributors such as (6Z)-nonen-1-ol and benzeneacetic acid, ethyl ester were upregulated, enhancing fruity and minty notes		^21^
3	274	Furans, pyrazines, ketones, and aldehydes	Liberica coffee (Indonesia)	Roasted coffee beans and brewed coffee	DVB/CAR/PDMS; 60 °C extraction for 20 min; HS–SPME–GC–MS	Roasting and brewing	Medium roasting yielded higher concentrations of dominant volatile groups, specifically furans, pyrazines, and ketones		^42^
4	257	Esters, hydrocarbons, heterocyclic compounds, terpenoids, aldehydes, alcohols, ketones, and aromatics	Arabica coffee (China)	Green coffee beans	DVB/CWR/PDMS; 60 °C extraction for 15 min; HS–SPME–GC–MS	Processing influence	Identified 13 key aroma compounds contributing to “mellow,” “fruity,” and “floral” flavor profiles		^5^
5	125	Aldehydes, alcohols, furans, carboxylic acids, pyrazines, pyridines, ketones, phenols, and esters	Arabica coffee (Colombia, Ethiopia, Brazil, Kenya, Tanzania, and Guatemala)	Green and roasted specialty coffee beans	DVB/CAR/PDMS; 75 °C extraction for 20 min (optimized via D-optimal design); HS–SPME–GC–MS	Origin authentication	Geographical origin and post-harvest processing were effectively discriminated using markers like 1-hexanol, nonanal, and d-limonene		^1^
6	82	Alcohols, aldehydes, esters, furans, furanones, ketones, organic acids, pyrazines, pyridines, pyrroles, sulfides, and terpenes	Arabica coffee (Thailand)	Ground roasted coffee beans	DVB/CAR/PDMS; 10 min equilibration at 40 °C followed by 20 min adsorption; HS–SPME–GC–MS	Shelf stability	Higher storage temperatures (50 °C) led to substantial quality loss through the degradation of compounds associated with fruity and floral notes		^12^
7	26	Pyrazines, furans, pyridines, alcohols, and organic acids	Arabica coffee (Australia)	Green and roasted coffee beans	PDMS/DVB; 40 °C incubation 15 min and extraction 15 min; HS–SPME–GC–MS	Fermentation	Specific pyrazines, such as methylpyrazine and 2-ethyl-5-methylpyrazine, were identified as markers to discriminate fermented from unfermented roasted coffee		^39^
8	23	Aldehydes, terpenes, alcohols, ketones, ethers, and volatile acids	Arabica (Ethiopia)	Green coffee beans	PDMS/DVB and CAR/PDMS; 30 °C extraction for 90 min; HS–SPME–GC–MS	Traceability analysis	High-altitude environments were associated with higher hundred-bean weight, moisture, and increased levels of chlorogenic acid, trigonelline, and sucrose		^6^
9	45	Pyrazines, furans, phenols, aldehydes, alcohols, esters, ketones, pyrroles, and thiophenes	Arabica coffee (Colombia)	Cold brew coffee	DVB/CAR/PDMS; 37 °C extraction for 50 min (5 min conditioning); HS–SPE and HS–SPME–GC–MS	Cold brew analysis	Key aroma contributors included linalool (floral/citrus), furaneol (caramel/sweet), and various pyrazines (chocolate/toasted)		^14^
10	31	Terpenes, esters, organic acids, pyrazines, furans, and ketones	Arabica coffee (Nicaragua)	Green coffee beans, roasted coffee beans, and immersion-brewed coffee	DVB/CAR/PDMS; 15 min equilibration followed by 45 min extraction at 50 °C; HS–SPME–GC–MS	Genetic aroma profiling	This study established a causal link between a specific genomic trait and the high-value sensory attributes of specialty coffee		^43^
11	87	Alcohols, phenols, ketones, pyrroles, acids, esters, aldehydes, pyrazines, pyridines, and furans	Arabica (China)	Cold brew coffee liquid	DVB/CAR/PDMS; 20 min equilibration at 30 °C, extraction at 40 °C; HS–SPME–GC–MS	Extraction optimization	Volatile profiles were comparable between methods, though UE coffee exhibited a slightly fruitier and nuttier aroma due to higher OAVs for specific markers like furan, 2-pentyl-		^2^
12	359	Pyrazines, aromatic hydrocarbons, phenols, ketones, aldehydes, furans, and pyrroles	Arabica coffee (Burundi, Rwanda, and Uganda)	Ground roasted coffee beans	DVB/CAR/PDMS; 60 °C for 30 min; HS–SPME–GC×GC-TOF-MS	Defect detection	Elevated levels of specific markers, such as 2,4-di-tert-butylphenol, suggest that microbial activity following insect damage is a primary pathway for the defect	>	^20^
13	14	Pyrazines, pyridines, organic acids, furans, ketones, and phenols	Arabica coffee (Panama, Australia)	Brewed espresso coffee	PDMS/DVB; 60 °C extraction for 15 min; HS–SPME–GC–MS; combined with portable NIR and a low-cost sensor e-nose	Processing and grinding	Volatile compound extraction efficiency was negatively correlated with grinding size, with finer particles (250 µm) yielding the highest concentrations		^38^
14	31	Alkaloids (caffeine, trigonelline), organic acids, pyrazines, pyridines, aldehydes, alcohols, and phenols	Arabica coffee (China)	Green coffee beans and medium-roasted coffee	DVB/CAR/PDMS; 60 °C extraction for 30 min; HS–SPME–GC–MS	Flavor improvement strategy	Anaerobic germination significantly reduced caffeine content by up to 62.25% and acrylamide formation in roasted beans by over 70%		^29^
15	51	Alcohols, acids, esters, aldehydes, ketones, alkanes, terpenes, pyrazines, pyridines, furans, pyrroles, and alkaloids	Arabica coffee (18 countries in Asia, Africa, Central, and South America)	Milled green coffee beans and roasted coffee beans	DVB/CAR/PDMS; 10 min incubation followed by 30 min extraction at 50 °C; HS-SPME–GC–MS	Origin discrimination	Natural processed coffees exhibited higher concentrations of linalool and 2,3-butanediol, whereas washed varieties were characterized by higher levels of hexanal		^10^
16	228	Esters, alcohols, hydrocarbons, phytosterols, sulfurous compounds, ketones, and aldehydes	Arabica coffee (Puerto Rico)	Healthy and brocaded coffee berries	DVB/CAR/PDMS; HS–SPME (60 °C incubation for 10 min, 30 min extraction) and DI-SPME (60 °C for 5 min); GC–MS	Ripening and infestation	The study established a comprehensive chemical “fingerprint” that identifies yellow berries as the most attractive stage for the pest due to their complex volatile emission		^55^
17	157	Alcohols, aldehydes, esters, alkanes, aromatic hydrocarbons, benzenes, ketones, pyrazines, and terpenes	Arabica coffee (Amazonas and Cajamarca)	Fermented green coffee beans	DVB/CAR/PDMS; 15 min equilibration and 45 min extraction at 50 °C; GC–MS	Fermentation comparison	Significant differences were found in 14 key compounds, including benzaldehyde, methional, and phenylethyl alcohol, which are linked to superior aroma quality		^56^
18	1212	Ketones, pyrazines, furans, aldehydes, esters, pyridines, carboxylic acids, alcohols, and amines	Arabica coffee (Brazilian states: Minas Gerais, São Paulo, Espírito Santo, Bahia, and Paraná)	Roasted and ground coffee beans	DVB/PDMS; 10 min equilibration followed by 20 min extraction at 52 °C; GC–MS	Origin discrimination	Altitude significantly influenced aroma quality; higher altitudes (G3, average 969.5 m) were associated with a more diverse and remarkable aroma, while lower altitudes (G1, average 618.5 m) yielded lower aromatic quality		^36^
19	75	Alkaloids, diterpenes, phenolic acids, organic acids, fatty acids, pyrazines, furans	Civet (*Luwak*) coffee (Indonesia)	Roasted powdered coffee	DVB/CAR/PDMS; incubation 45 min at 60 °C, equilibration at 28 °C;HS–SPME–GC–MS	Premium authentication	*Luwak* coffee contained significantly lower levels of caffeine (2.85 µg/mg) and trigonelline (0.14 µg/mg) than conventional roasted Arabica		^37^
20	234	Furans, pyrazines, pyridines, pyrroles, acids, alcohols, aldehydes, and esters	*Coffea arabica* beans (Brazil, Ethiopia, Colombia, and Indonesia)	Ground roasted coffee beans	DVB/CAR/PDMS; 10 min equilibration followed by 20 min extraction at 40 °C; HS–SPME–GC–MS	Roasting duration effects	Increasing roasting time from 11 to 14 minutes significantly reduced concentrations of pyridine and 2-furanmethanol acetate, while increasing levels of 5-methyl-2-furancarboxaldehyde		^30^
21	83	Sulfur compounds/thiols, furans, pyrazines, heterocyclic compounds, phenolics, aldehydes, ketones	Italian espresso blend (Central/South America and East Africa)	Ground roasted coffee powder and brewed espresso beverage	PDMS/DVB; extraction at 50 °C for 40 min (powder) or 5 min (beverage); HS–SPME–GC–MS	Roasting intensity impact	Furfuryl acetate and furfural were identified as effective analytical markers for the strength of the roasting process		^8^
22	7	Furanic compounds, aldehydes (3-methylbutanal, hexanal), and acrylamide	Arabica (India)	Ground roasted coffee beans	DVB/C-WR/PDMS; 15 min incubation and 30 min extraction at 60 °C; HS–SPME–GC–MS	Species and processing effects	Contrary to typical findings, Arabica samples in this study exhibited significantly higher acrylamide content than Robusta		^19^
23	39	Pyrazines, pyridines, furans, thiazoles, terpenes, aldehydes, alcohols, ketones, pyrroles, alkaloids, and phenolic acids	Arabica coffee (Brazil)	Cold brew coffee extracts	DVB/CAR/PDMS; 60 °C (water bath) for 20 min; HS–SPME–GC–MS	Quality control markers	Peak caffeine concentrations (603–631 mg/L) were achieved using beans roasted at 220 °C		^41^
24	63	Furans, pyrazines, phenols, pyrroles, pyridines, aldehydes, ketones, phenolic acids (e.g., chlorogenic acid), and flavonoids	Arabica coffee (China and Nigeria)	Roasted ground seeds/beans and aqueous brews	DVB/CAR/PDMS; 60 °C for 30 min (10 min incubation); HS–SPME; GC–MS	Coffee substitute evaluation	Baobab seeds are caffeine-free and provide sensory profiles comparable to coffee, particularly in roasted and nutty notes		^46^
25	108	Pyrazines, pyridines, furans, pyrroles, alcohols, acids, aldehydes, esters, and alkaloids	Specialty Arabica coffee (Brazil)	Roasted ground coffee powder	DVB/CAR/PDMS; 20 min equilibration and 30 min extraction at 60 °C; GC–MS	Roasting process optimization	Key chemical markers for profile discrimination included furfural, furfuryl alcohol, pyridine, and 4-vinylguaiacol		^7^
26	62	Pyrazines, furans, ketones, acids, furanones, pyrroles, aldehydes, pyrones, phenols, esters, pyridines, lactones, and alcohols	Arabica coffee (Colombia)	Roasted and ground coffee powder	CAR/PDMS; 30 min extraction at 60 °C; HS–SPME–GC–MS	Fermentation comparison	The natural process achieved the highest sensory score (86.63) and was statistically distinct from other methods		^33^
27	29	Furans, pyrazines, pyridines, pyrroles, furanones, acids, aldehydes, alcohols, esters, phenols, and thiophenes	Arabica coffee (Brazil)	Roasted and ground coffee beans	DVB/CAR/PDMS; 70 °C for 30 min; HS–SPME–GC–MS	Sensory prediction modeling	Identified 15 volatile compounds as primary chemical markers responsible for predicting coffee quality		^11^
28	138	Furans, pyrazines, esters, pyrroles, pyridines, aldehydes, ketones, phenols, sulfur compounds, and carbonic acids	Single-origin Arabica (Brazil, Colombia, El Salvador, Ethiopia, Honduras, Mexico, Papua New Guinea, and Peru)	Roasted ground coffee	CAR/PDMS; 10 min equilibration and 30 min extraction at 50 °C; HS–SPME–GC–MS	Roasting customization	Roasting degree was the primary factor for sample differentiation, overriding geographical origin in chemometric clustering		^32^
29	155	Pyrazines, phenols, pyrroles, sulfur compounds, chlorogenic acid derivatives, and alkaloids	*Coffea arabica* and *Coffea canephora* (Italy)	Roasted ground coffee powder and filter machine brew	PDMS/DVB; 40 min extraction at 50 °C with vibration; HS–SPME–GC–MS and LC-UV/DAD	Industrial quality prediction	Volatile fingerprints (aroma) were the primary drivers of sensory perception and were sufficient to predict even “taste” attributes like bitterness and acidity		^16^
30	198	Furans, pyrazines, pyridines, pyrroles, furanones, aldehydes, ketones, alcohols, phenols, terpenes, fatty acids, and alkaloids	Arabica coffee (Brazil)	Roasted ground coffee powder	DVB/CAR/PDMS; 30 min extraction at 70 °C; HS–SPME–GC–MS	Processing comparison	Five primary markers (2-furylmethanol, octadecanal, 2-acetyl-3-methylpyrazine, DDMP, and caffeine) were identified as the most significant contributors to mathematical quality models		^45^
31	47	Aromatic heterocycles (furans, pyrazines, pyrroles, pyridines, pyrans), phenols, aldehydes, ketones, alcohols, esters, lactones, and fatty acids	Philippine Arabica and Robusta (Cordillera, Kalinga, Asipulo, and Matutum)	Roasted whole coffee beans	DVB/CAR/PDMS; 10 min equilibration and 20 min extraction at 70 °C; HS–SPME–GC–MS	Geographical classification	Principal Component Analysis (PCA) successfully discriminated Arabica from Robusta based on higher levels of acetic acid, furfural, 5-methylfurfural, 2-formylpyrrole, and maltol in Arabica samples		^13^
32	3	3-Alkyl-2-methoxypyrazines	Arabica and Robusta (Africa, Asia, Central America, and South America)	Green coffee beans	CAR/PDMS; 30 min extraction at 60 °C; HS–SPME–GC–MS (SIM mode)	Defect reference criteria	3-Isopropyl-2-methoxypyrazine was identified as the primary chemical marker for PTD, showing concentrations up to 1,000 times higher in defective/insect-damaged beans than in wholesome samples		^34^
33	74	Furans, pyrazines, aldehydes, ketones, pyrroles, esters, pyridines, sulfur compounds, phenolic compounds, and terpenes	65 commercial espresso capsules (Italy)	Capsule-brewed espresso coffee	PDMS; 60 °C for 25 min (following 5 min equilibration); HS–SPME–GC–MS	Brand fingerprinting	Volatile profiles generally showed low variability within the same brand, with significant differences observed primarily in samples containing flavor additives like caramel or chocolate		^22^
34	11	Furanoids (furans), non-furan heterocyclic compounds (pyrazines, pyrroles), and benzene derivatives	Ethiopian Yirgacheffe coffee (Ethiopia)	Roasted whole beans, ground powder, and coffee infusions	DVB/CAR/PDMS; 5 min equilibration and 30 min extraction at 70 °C; GC–MS	Storage optimization	*N-furfuryl* pyrrole served as a key positive marker for flavor quality, while 2,4-bis(1,1-dimethylethyl)-phenol was the primary negative indicator of aroma		^31^

## Data synthesis workflow

This review provides a quantitative greenness assessment focused on sample preparation steps used for the determination of VOCs in coffee matrices. Greenness evaluation was performed using AGREEprep, a metric specifically developed to measure the environmental impact of the sample preparation stage independently of instrumental detection. AGREEprep was selected as the primary greenness metric because it is a tool designed specifically for the evaluation of sample preparation. Alternative tools assess different stages of the analytical workflow. For example, the green analytical procedure index (GAPI)^24^ and its more recent extension ComplexGAPI^25^ and ComplexMoGAPI^26^ evaluate the entire analytical procedure, including instrumental analysis, reagent toxicity, and pre-analytical steps, but treat sample preparation as a single component. The analytical greenness metric (AGREE)^27^ follows the twelve principles of green analytical chemistry and also covers the complete procedure rather than focusing on the extraction step. In this review, SPME methods share the same downstream analytical stage (GC-MS in most studies), so a metric centered on sample preparation provides a more discriminating comparison among the included studies.

### PRISMA-based selection and eligibility criteria for AGREEprep assessment

**Fig. 1:**
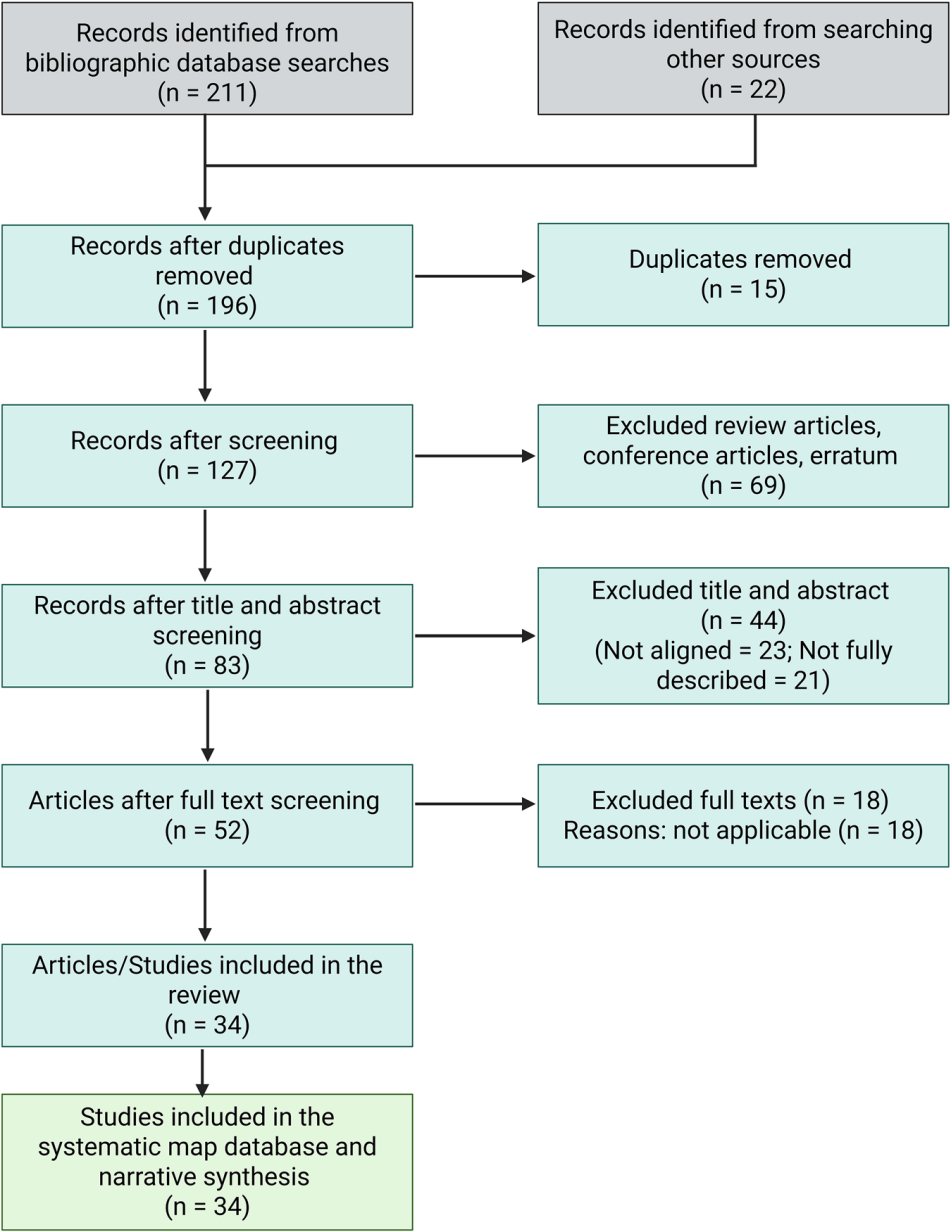
PRISMA flow diagram illustrating study identification for coffee analysis with SPME. (Based on ref. ^28^).

To identify the studies included in this review, a structured literature search was conducted in Scopus, Web of Science, ScienceDirect, and PubMed, covering peer-reviewed original studies published between 2020 and 2025 reporting experimental coffee volatile profiling using SPME and describing the sample preparation workflow in sufficient detail for AGREEprep evaluation. The overall study design followed a PRISMA-guided workflow to ensure transparent identification, screening, eligibility assessment, and final inclusion of studies in the scoring dataset. After initial retrieval, records were screened for relevance to coffee quality assessment using SPME-based volatile profiling and then refined based on AGREEprep applicability, considering that AGREEprep requires explicit methodological information for the sample preparation steps. Inclusion criteria required (i) original experimental research on coffee volatile compound analysis using SPME; (ii) a described sample preparation procedure; and (iii) availability of quantitative or stated qualitative information enabling evaluation of key AGREEprep criteria, including solvent/reagent identity and volumes, number of discrete preparation steps (equilibration, extraction, desorption), total preparation time, energy-relevant conditions (temperature requirements), and waste generation attributable to the preparation stage. Exclusion criteria comprised absent or undefined SPME parameters, insufficient reporting of extraction conditions (temperature, time, fiber type), purely qualitative descriptions preventing reproducible scoring, studies employing non-SPME extraction techniques (LLE, SPE, SAFE), and non-experimental publications (reviews, modelling-only works, conference abstracts) (see Fig. 1), based on a systematic literature search methodology described by Schnarr et al.^28^. At the full-text screening stage, studies were excluded if critical AGREEprep parameters were not applicable due to insufficient methodological detail.

### Bibliometric overview of the selected dataset

**Fig. 2:**
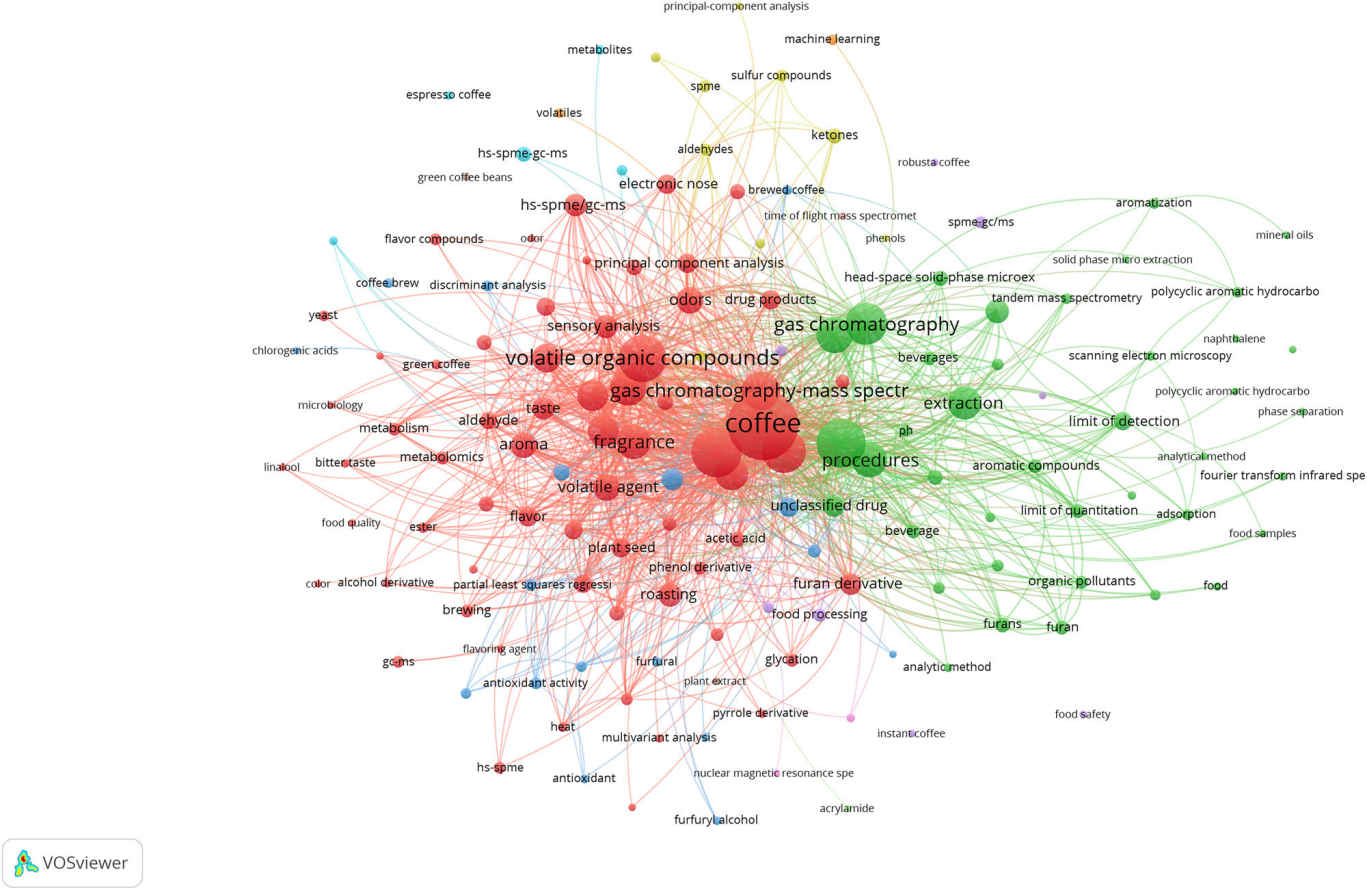
Bibliometric keyword co-occurrence map of Scopus-indexed articles (2020-2025) using VOSviewer (version 1.6.20). In this map, each node (circle) represents a keyword that appeared in the analyzed publications, and lines between nodes indicate that two keywords were used together in the same article. The larger a node, the more frequently that keyword appeared across the literature; the thicker a line, the more often two keywords co-occurred. Keywords are grouped into color-coded clusters based on how strongly they co-occur with one another, with each cluster representing a distinct research theme.

To characterize the thematic structure of the studies retained after PRISMA screening, a keyword co-occurrence map was constructed in VOSviewer from the Scopus-indexed records published between 2020 and 2025 (see Fig. 2). Four main research clusters are visible: (1) a red cluster, the largest, centered on coffee, volatile organic compounds, aroma, and fragrance, representing studies on coffee flavor chemistry and sensory analysis; (2) a green cluster focused on analytical extraction and detection methods, including gas chromatography, extraction procedures, and solid-phase microextraction; (3) a yellow cluster representing chemometric and data-driven approaches such as principal component analysis, machine learning, and SPME variants (HS-SPME, HS-SPME-GC-MS); and (4) a blue cluster, a smaller group associated with non-volatile compounds and biological markers including antioxidant activity and chlorogenic acids.

### AGREEprep scoring methodology

The AGREEprep metric evaluates ten criteria of the sample preparation workflow such as (1) sample preparation location, (2) use of safer reagents and solvents, (3) sustainable, reusable, and renewable materials, (4) waste production, (5) sample, chemical, and material amounts, (6) sample throughput, (7) process steps or automation, (8) energy consumption, (9) post-sample preparation configuration, and (10) number of hazards. Each criterion is assigned penalty points (0–0.17) based on predefined scoring rules, with lower penalties indicating greener practices. The total AGREEprep score is calculated as 1 minus the sum of penalty points, yielding values between 0 (least green) and 1 (most green)^27^. The studies *(n* = *34)* were evaluated using AGREEprep, and the results were recorded in an Excel template (see Table 1 and **Supplementary Material**).

## Solid-phase microextraction techniques applied to coffee

**Fig. 3:**
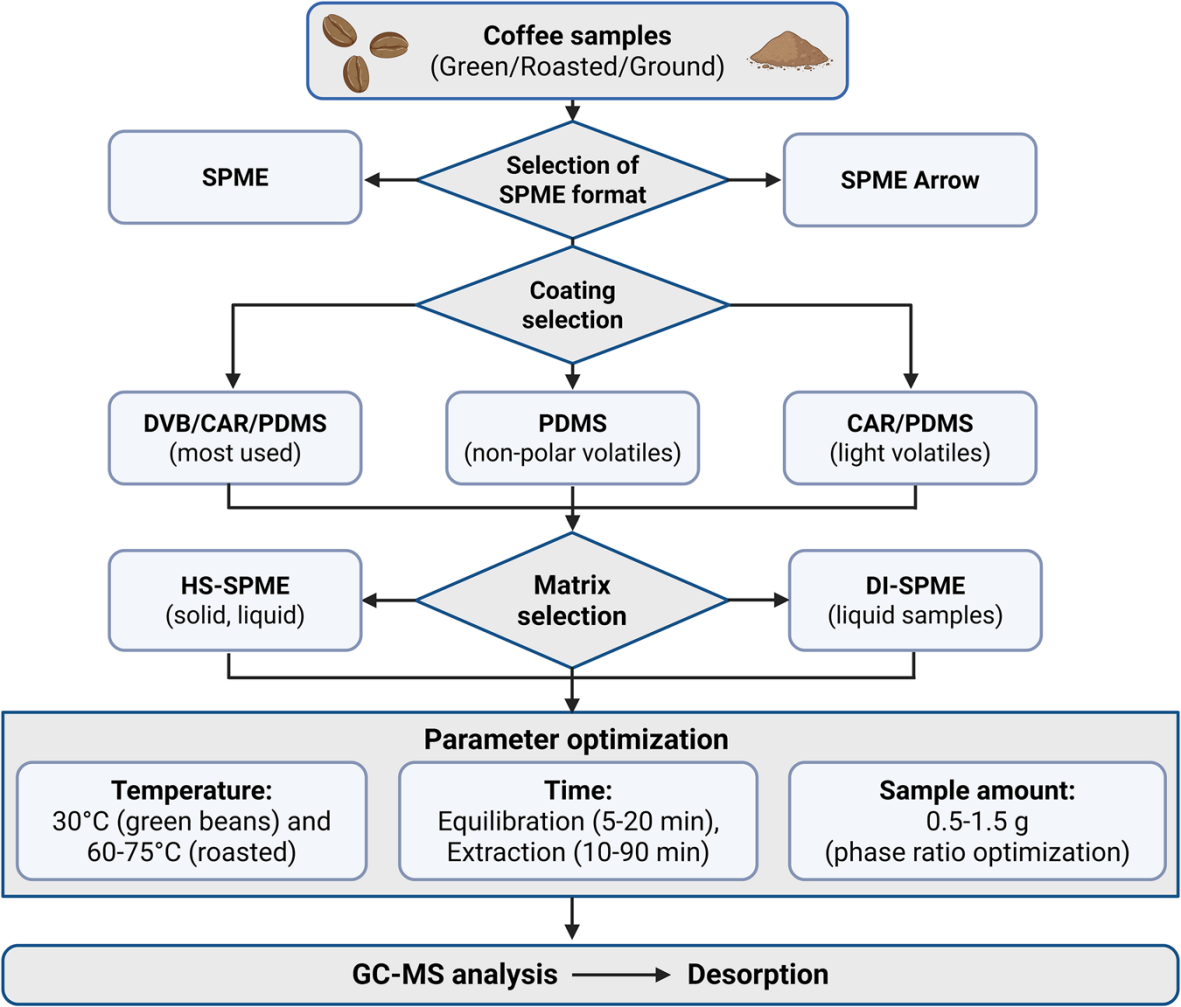
SPME workflow for the analysis of coffee VOCs. The decision tree is organized in sequential steps. (i) selection of the SPME format, and (ii) selection of the coating. Both SPME formats accept the same range of coatings. Subsequent steps include the choice of extraction mode (HS-SPME or DI-SPME), optimization of extraction parameters (temperature, equilibration time, extraction time, sample amount), thermal desorption, and GC-MS analysis. The figure shows the most frequent combinations reported in coffee VOC studies.

The SPME workflow for coffee analysis includes five main steps, as shown in Fig. 3: (i) selection of the SPME format (conventional fiber SPME or SPME Arrow), (ii) selection of the coating, (iii) matrix selection, (iv) parameter optimization, and (v) GC–MS analysis.

The choice of SPME format is made first, because it defines the geometry, sorbent volume, and mechanical robustness of the extraction device. Conventional fibers are widely used because of their established protocols, lower cost, and compatibility with most commercial autosamplers. SPME Arrow, which carries a sorbent phase about five to twenty times larger than that of a conventional fiber and has a closed tip that protects the coating during septum penetration, is preferred for trace-level analysis and for matrices where mechanical stability matters. After the format has been chosen, the coating is selected on the basis of the volatility and polarity of the target compounds (see Table 2). Both conventional fibers and SPME Arrow are commercially available with the same range of coatings, including DVB/CAR/PDMS, divinylbenzene/carbon-wide-range/polydimethylsiloxane (DVB/C-WR/PDMS), CAR/PDMS, PDMS/DVB, DVB/PDMS, and PDMS. In most coffee VOC studies, DVB/CAR/PDMS is used for broad volatile profiling, whereas CAR/PDMS is more appropriate for light volatile compounds. Next, the extraction mode is chosen according to the physical state of the sample. Headspace SPME (HS–SPME) can be used for both solid and liquid samples, while direct immersion SPME (DI-SPME) is limited to liquid samples. At the same time, key extraction conditions should be optimized, including extraction temperature, equilibration time, extraction time, and sample amount. For green coffee beans, a lower extraction temperature of about 30 °C is usually selected to avoid thermal degradation. In contrast, roasted coffee is commonly extracted at 60 to 75 °C. Equilibration time usually ranges from 5 to 20 min, whereas extraction time ranges from 10 to 90 min. The sample amount is generally kept between 0.5 and 1.5 g to obtain a suitable phase ratio. After extraction, the enriched volatile compounds are thermally desorbed into the GC–MS system, where they are separated and identified. Optimal sensitivity, selectivity, and reproducibility require systematic optimization of experimental conditions. This requirement arises because extraction efficiency depends on thermodynamic and kinetic equilibrium between the fiber coating and the coffee matrix. Therefore, careful control of these variables is necessary. The following sections describe the main parameters that influence SPME performance in coffee analysis, including fiber selection, extraction temperature, extraction time, and sample mass and volume.

**Table 2:** Typical applications and representative volatile analytes extracted by different SPME fiber coatings

Type of fiber	Main use	Representative target analytes	Ref.
DVB/CAR/PDMS	Broad-range extraction of volatile compounds with diverse polarities and molecular weights	Hexanal, octanal, 2-methylpyrazine, ethylpyrazine, furfural, benzaldehyde, linalool, limonene	^1,7,12,15,20,30,31,38–40,46,56^
PDMS/DVB	Extraction of moderately polar aromatic and semi-volatile compounds	Pyridine, methylpyrazine, 2,5-dimethylpyrazine, benzaldehyde, phenylethyl alcohol, methyl salicylate	^8,9,16,38–40^
CAR/PDMS	Extraction of small and highly volatile compounds due to microporous adsorption properties	2-methylfuran, 2,5-dimethylpyrazine, acetylpyrazine, 2-furanmethanol, 4-vinylguaiacol	^32,33^
DVB/PDMS	Extraction of semi-volatile compounds with medium polarity	2,3-butanedione, 2,3-pentanedione, 2-methylpyrazine, furfural, acetic acid	^36^
PDMS	Extraction of non-polar volatile compounds through absorption mechanism	Limonene, β-pinene, p-cymene, terpinolene, nonanal, octanal	^22^

### Key optimized parameters for SPME

#### Fiber selection

The SPME format selected in the previous step requires an appropriate SPME coating, since it determines which VOCs can be detected and how sensitive the analysis is to different chemical compounds^14,19^. Among commercially available coatings, DVB/CAR/PDMS fibers are most extensively applied in coffee VOCs (see Table 2), commonly at 50/30 µm or 75 µm thicknesses for roasted beans and brewed infusions^7,12,20,29–31^. Their broad sorptive capacity enables simultaneous extraction of low-molecular-weight volatiles and semi-volatile aroma compounds, resulting in comprehensive chromatographic fingerprints^14^.

A modified variant, DVB/C-WR/PDMS^19,21^, has demonstrated improved extraction efficiency for ground coffee volatiles compared with conventional DVB/PDMS^8,16^ or PDMS^14,22^ fibers and has been applied to differentiate fermentation-driven aroma profiles in Arabica coffees. The development of SPME Arrow geometries represents a significant technological advancement, offering a five- to twenty-fold larger sorbent phase than conventional fibers. This increased capacity enhances sensitivity for trace-level markers relevant to origin authentication, processing differentiation, and defect detection. Although triple-coated fibers provide the broadest chemical coverage, dual- and single-phase fibers are employed for targeted selectivity or comparative evaluations. PDMS/DVB (65 µm) is frequently used for fingerprinting roasted powders^14,19^, espresso capsules, and cold brew samples, offering balanced representation of major volatiles. CAR/PDMS^6,32–34^ (75 µm) is effective for small, highly volatile compounds such as methoxypyrazines associated with the potato taste defect. In contrast, DVB/PDMS and single-phase PDMS fibers exhibit narrower selectivity, with PDMS favoring non-polar analytes and generally yielding lower total peak areas in complex coffee headspace matrices^22^. Systematic comparisons consistently identify triphasic DVB/CAR/PDMS as the most suitable coating for comprehensive coffee profiling, owing to its ability to capture a wide polarity range from highly volatile alcohols and aldehydes to semi-volatile esters and aromatic compounds. The integration of these multiphase coatings with SPME Arrow technology further improves detection limits and analytical robustness, reinforcing their utility in advanced coffee quality assessment and authenticity studies^15^.

Before initial use, SPME fibers must undergo thermal conditioning, usually at 270 °C for 60 min, to remove residual compounds from the manufacturing process. In addition, routine re-conditioning between analyses, commonly for 10 min at 250 to 270 °C, is required to prevent carryover and maintain a stable baseline. From the perspective of green chemistry, the repeated use of these devices is a major advantage. For example, commercial coatings such as DVB/CAR/PDMS can be used for more than 200 analytical cycles without major loss of performance. As a result, one fiber can replace many disposable SPE cartridges. This feature reduces both hazardous waste and consumable costs, particularly in high-throughput laboratories. Furthermore, advanced formats such as SPME Arrow can prolong service life because of their greater mechanical stability and higher sorbent capacity. Taken together, these features support the use of SPME as a sustainable and cost-efficient platform for routine coffee quality control.

#### Extraction temperature

Temperature is a critical thermodynamic parameter that modulates the partition coefficients of analytes between the phases^9,15^. Higher temperatures (typically 50 °C to 75 °C) accelerate the extraction rate and increase the concentration of semi-volatiles in the headspace. They may also carry the risk of inducing thermal degradation or forming flavor artifacts that are not present in the original matrix^1,20,35^. Specifically, research on regional authentication has demonstrated that an adsorption temperature of 70 °C yields a higher number of volatile features (408 vs. 380 compounds at 50 °C) and more reliable fingerprints for classifying coffee origins^15^. Conversely, lower temperatures near 30 °C are often employed during the fingerprinting of green coffee beans to prevent the induction of Maillard-type artifacts that could obscure the raw bean’s chemical identity^9^. Furthermore, excessively high temperatures may result in a displacement effect, where high-boiling compounds occupy the fiber’s active sites to the detriment of substances with high vapor pressure^13^.

#### Extraction time

As a kinetic process, SPME requires a specific duration to reach a state of equilibrium between the VOCs and the fiber coating^14,19^. In coffee VOC analysis, extraction times vary based on the matrix, typically ranging from 10 to 90 minutes^6,19^. Optimization studies for complex matrices like cold brew coffee have identified an asymptotic behavior in extraction yields, where a plateau is reached at approximately 46–50 minutes. Exceeding this duration provides no significant improvement in the number of detected peaks^14^. There is an established inverse relationship between extraction time and the volatility of analytes. Shorter durations (typically 10 min) favor low-boiling volatiles, whereas extended periods (30–50 min) are necessary for the recovery of semi-volatile compounds from roasted grounds^20^. However, excessive extraction times can lead to competitive displacement, where analytes with lower sorbent affinity are desorbed by compounds with higher affinity.

#### Sample mass and volume

The quantity of sample utilized is a decisive factor in determining the phase ratio (β), defined as the ratio of the headspace volume to the sample volume^13^. For roasted coffee beans, increasing the sample mass from 0.5 g to 1.0 g has been shown to substantially improve peak intensities, yet a further increase to 1.5 g often fails to yield additional responses due to a decrease in the phase ratio and the exhaustion of the fiber’s retention capacity^13^. For liquid coffee infusions, an optimal sample volume of 6 mL within a 20 mL vial is frequently established to maintain sufficient headspace for efficient volatilization while preventing fiber fouling through direct contact with the liquid^14^. Such contact can introduce non-volatile compounds into the fiber coating, thereby altering the accurate volatile composition and decreasing the effective headspace.

### Advanced analytical techniques for VOCs separation in coffee analysis

GC-MS is still the main analytical tool used to study the complex VOCs in coffee (see Table 1)^7,11,30,36^. Its widespread use in research and industrial quality control is due to its high sensitivity, excellent reproducibility, and the availability of extensive spectral libraries, including NIST and Wiley, which facilitate the definitive identification of a broad spectrum of VOCs^7,19,29^. This methodology is routinely employed to monitor both sensory-related compounds and safety-related contaminants, such as furanic derivatives and polycyclic aromatic hydrocarbons^22,29^. To enhance sensitivity for trace-level analytes or specific biomarkers like methoxypyrazines associated with the potato taste defect, the utilization of selected ion monitoring mode significantly lowers detection limits^5,21,34^. However, one-dimensional GC systems have limited peak capacity. As a result, co-elution often occurs in complex roasted coffee samples. In such cases, reliable compound assignment requires chemometric deconvolution together with retention index verification.

To address the peak capacity limitations of GC systems when analyzing more than 1000 compounds present in roasted coffee, comprehensive two-dimensional gas chromatography with time-of-flight mass spectrometry (GC×GC–TOFMS) is a high-resolution alternative^20,32^. Through thermal modulation, this platform connects two columns with complementary polarities, achieving a nearly 10-fold increase in peak capacity and substantially improved signal-to-noise ratios^20^. This enhanced resolution facilitates the identification of over 1000 analytes, providing a significantly deeper understanding of the volatile fingerprints related to geographical origin and quality defects^7,30^.

Column selection is also important for separation quality both in one- and two-dimensional GC–MS. Polar polyethylene glycol columns, such as DB-Wax and Rtx-Wax, are most often used because they provide better separation of polar volatile compounds, including alcohols, aldehydes, and acids. In contrast, non-polar stationary phases such as DB-5ms and HP-5ms are more suitable when the target analytes are of medium to low polarity. In coffee VOC analysis, this includes terpenes, alkyl-substituted aromatics, and long-chain aldehydes, which interact more efficiently with the methylphenyl-siloxane phase and therefore show sharper peaks and better resolution on these columns. The choice between polar and non-polar columns is therefore guided by the chemical nature of the targeted volatile fraction rather than by a generic preference for broad or narrow screening^2,36^.

For targeted quantification of specific safety markers, such as acrylamide, liquid chromatography coupled with triple quadrupole mass spectrometry provides superior selectivity and accuracy^19,30^. These systems help verify if coffee complies with regulatory limits (e.g., 400 μg/kg for roasted coffee) and help understand how compounds such as asparagine and reducing sugars contribute to the formation of toxic substances^5,29^. Furthermore, high-resolution mass spectrometry platforms, including Orbitrap and Q-Exactive systems, are increasingly utilized for untargeted metabolomic fingerprinting of both volatile and non-volatile fractions^8,21,37^. These systems offer precise mass accuracy and the capability for data-dependent acquisition, allowing for the unmasking of unique markers associated with premium varieties or specialized processing methods^1,6,9,13,16,21,36,37^.

Complementary to these laboratory-based systems, the development of portable digital technologies offers transformative potential for in situ quality assurance^1,12,38^. Miniature near-infrared (NIR) spectrometers allow for rapid, real-time monitoring of critical parameters such as moisture content, roasting intensity, and chemical precursors directly on coffee farms or within industrial production lines^2,12,38,39^. These digital tools can effectively predict extraction endpoints and differentiate cultivars based on unique absorption bands^2,40^. Concurrently, e-nose devices, which utilize diverse sensor arrays to mimic human olfaction, provide rapid and objective alternatives to traditional sensory panels for assessing aroma profiles and regional authentication^39,40^. However, the sensitivity of metal-oxide and conducting-polymer sensors typically lies in the µg L^−^^1^ to mg L^−1^ range, which is two to four orders of magnitude lower than that of HS-SPME-GC-MS for trace odorants. Sensor arrays also produce a global response to the headspace rather than identifying individual compounds, so the resulting signal reflects the overall odor pattern rather than the contribution of specific odor-active molecules. By contrast, gas chromatography-olfactometry (GC-O) combines chromatographic separation with a human sniffing port, allowing the identification of individual odor-active compounds and the estimation of their relative impact through aroma extract dilution analysis or odor activity values. GC-O is slower, requires trained assessors, and is less suitable for high-throughput screening, but it remains the reference technique for linking chemical composition to sensory perception. E-nose devices are therefore best understood as complementary screening tools rather than substitutes for chromatographic methods coupled with olfactometric detection. When integrated with advanced chemometric modeling and machine learning algorithms, such as artificial neural networks, these portable platforms establish a robust framework for high-throughput coffee quality classification and certification^1,22,39,40^.

## Coffee quality attributes assessed by SPME

### Aroma and flavor profiling

Coffee quality is conceptualized as a multidimensional construct centered on aroma, which integrates complex chemical and perceptual interactions^5,6^. As a result, establishing robust, data-driven correlations between molecular fingerprints and sensory perception has become a central objective in contemporary food science^9^. Such integration aims to complement, rather than replace, traditional cupping by providing reproducible chemical metrics that underpin sensory quality. More than 1000 VOCs contribute to the sensory profile of coffee, most of them formed during roasting through Maillard reactions, Strecker degradation, and sugar caramelization^12,19,22^. Flavor perception arises from the interaction between these volatile constituents perceived both orthonasally and retronasally and non-volatile bioactive compounds that stimulate gustatory receptors. Although coffee contains many different volatile compounds, only a small fraction (about 5%) has OAVs high enough to significantly influence aroma perception and value attribution^7^. This disproportion between chemical abundance and sensory relevance highlights the necessity for selective and sensitive analytical approaches capable of discriminating key odorants from the broader volatile background.

#### Key volatile classes and sensory impact

Furans and furanones constitute one of the most abundant volatile classes in roasted coffee, representing approximately 25–41% of total VOCs^19,32,38^. They are primarily associated with sweet, caramelized, roasted, and bread-like notes^19^. Among them, 2-furfurylthiol is widely recognized as a high-impact odorant contributing to the characteristic “freshly roasted” aroma^8,29^, whereas furfural imparts woody and almond-like nuances^8,32^. Although abundant, their sensory contribution is concentration-dependent and strongly influenced by roasting intensity^19^.

Pyrazines, accounting for roughly 25–39% of the volatile profile, provide foundational roasted, nutty, cocoa, and hazelnut notes^19,38,41,42^. Compounds such as 2-methylpyrazine and 2,5-dimethylpyrazine are used as indicators of roasting degree and, in some cases, as markers for origin differentiation^33,35,41^. In contrast, specific methoxypyrazines, most notably 2-isopropyl-3-methoxypyrazine, are associated with the potato taste defect, imparting undesirable earthy or vegetal off-flavors^20,34^. The characteristic aroma of roasted coffee arises from distinct chemical classes that undergo significant quantitative and qualitative changes during processing (see Table 3). This dual role highlights the fine balance between desirable and defective sensory outcomes within the same chemical class.

**Table 3:** Representative volatile classes and marker compounds in coffee and their sensory contributions

Volatile class/compound	Potential sensory contribution	Ref.
Pyrazines	Walnut, spicy, sweet corn, toasted	^14^
Furfural	Almond, bitter taste, earthy, roasty, burnt, and phenolic	^2,16^
Pyridine	Bitter, roasted, burnt, and smoky flavors, special odors, irritating odors	^2^
Furfuryl pyrrole	Earthy, roasty, burnt, and phenolic	^16^
Vanillin	Vanilla, sweet	^14^
Maltol	Sweet, caramellic, and roasted aroma and flavor	^39^
Linalool	Grassy, citrusy, sweet, fruity, and floral aroma	^44^
Limonene	Floral, fruity, and nutty aroma	^44^
3-methylbutanale	Almond, fruity	^22^
2-ethylpyrazine	Earthy, musty	^22^
2,3-Pentanedione	Sweet and floral	^44^
4-Vinylguaiacol	Roasty, burnt, and phenolic	^16^
Guaiacol	Phenolic, spicy	^22^
2-Isopropyl-3-methoxypyrazine	Earthy, vegetable, potato	^20^
Benzeneacetic acid	Anise and chocolate flavors	^56^

Aldehydes and ketones contribute fruity, green, malty, buttery, or creamy attributes. For example, 3-methylbutanal is recognized as a key odorant associated with malty notes, while hexanal serves as a sensitive marker of lipid oxidation and storage-induced staling^12,19,42^. Ketones such as 2,3-butanedione and 2,3-pentanedione are critical for buttery and creamy characteristics but may become excessive under certain processing conditions^2,36,42^. Their dynamic formation and degradation during roasting and storage further complicate quality assessment.

Phenolic compounds, derived primarily from chlorogenic acid degradation, impart smoky, spicy, and clove-like nuances^12^. High-impact phenols such as 4-vinylguaiacol and guaiacol can enhance aromatic complexity at moderate concentrations but may dominate the profile in darker roasts, potentially masking subtler attributes. This concentration-dependent behavior reinforces the importance of quantitative profiling.

Terpenes, including limonene and linalool, are associated with premium sensory profiles characterized by citrus and floral notes^5,12,42,43^. Their relative abundance is influenced by genotype and processing conditions. Recent transcriptomic studies have identified specific terpene synthases as key contributors to the pronounced floral character of the “Geisha” variety, linking genetic determinants with distinctive sensory phenotypes^43^. While such findings deepen mechanistic understanding, broader validation across cultivars and environments remains necessary to confirm their consistency as authenticity markers.

### Roasting degree and process monitoring

Roasting constitutes the key thermal transformation in the coffee production chain, converting chemically stable green beans into a structurally and aromatically complex system through Maillard reactions, Strecker degradation, caramelization, and pyrolysis^8,12,42^. The extent and kinetics of these reactions are governed by time–temperature profiles, which critically modulate both volatile and non-volatile fractions. Indeed, roasting degree frequently exerts a stronger influence on the resulting molecular signature than geographical origin or botanical variety^12,32^, making roasting a key factor in defining the sensory identity of coffee. From an industrial perspective, this technological predominance needs rigorous process control supported by reliable chemical markers to ensure sensory consistency, product standardization, and compliance with safety regulations^19^.

A suite of roasting-dependent molecular indices has therefore been proposed to monitor thermal progression. Among toxicological markers, acrylamide, classified as a Group 2 A probable carcinogen, holds regulatory relevance. Its concentration typically reaches maximum levels in light roasts and decreases with increasing roast intensity due to thermal degradation above approximately 175 °C^19^. In contrast, certain furanic derivatives, such as 5-hydroxymethylfurfural, are associated with advanced heat exposure; however, their concentration trends may exhibit non-linear or oscillatory behavior across roasting stages. These contrasting kinetic patterns point out the limitations of relying on single-compound indicators and highlight the need for integrated chemical profiling^44,45^.

Heterocyclic compounds further reflect roast development. Pyridine, generated primarily through the thermal decomposition of trigonelline, is widely recognized as a marker of dark roasting^13,41^. Although its concentration increases with roast severity, excessive accumulation correlates with burnt and bitter sensory attributes, illustrating the delicate balance between desirable roast character and quality deterioration^10,12^. Pyrazines such as methylpyrazine and 2,5-dimethylpyrazine are initially formed via Maillard pathways and contribute roasted, nutty, and cocoa notes. However, prolonged exposure to high temperatures may induce secondary degradation, complicating their interpretation as linear indicators of roasting intensity^42^.

Thermal degradation also affects acidic and bioactive precursors. Chlorogenic acids, which contribute to perceived acidity and cup “brightness,” progressively decrease with increasing roasting temperature, beginning at relatively early stages of heat treatment^6,8,42^. Acetic acid exhibits a similar declining trend due to volatilization and decomposition^13,40^. In contrast, caffeine remains comparatively thermostable, displaying minimal concentration changes across roast degrees; this relative stability supports its use as a normalization reference in quantitative models, although matrix-specific variability should be acknowledged^6,35^.

Complementary physical metrics, particularly colorimetric parameters, provide rapid and non-destructive indicators of roast progression. Decreases in lightness and yellowness correspond to the accumulation of high-molecular-weight melanoidins formed during advanced Maillard reactions^19,42,46^. While such physical measurements offer operational utility in industrial settings, they lack molecular specificity and therefore benefit from integration with chemical analytics^12,46^.

### Freshness, storage, and shelf-life

Freshness and shelf-life assessment are important for coffee quality, because the volatile balance formed during roasting is naturally unstable. Post-roast sensory decline, commonly referred to as staling, reflects the simultaneous loss of desirable aroma compounds and the formation of oxidation-derived off-flavors^31^. This process includes volatilization, lipid oxidation, and other changes in compounds formed during roasting. HS-SPME coupled with GC–MS provides a non-destructive and time-resolved analytical framework to interrogate these stability kinetics, enabling objective tracking of compositional shifts without extensive sample manipulation^6^.

Identifying reliable molecular markers is important to relate chemical degradation to measurable quality indicators. Hexanal is widely recognized as a primary indicator of lipid oxidation, formed via oxidative cleavage of unsaturated fatty acids^12,19^. Its concentration increases during storage and correlates with earthy, bitter, and rancid sensory attributes. However, reported oscillatory behavior suggests that hexanal alone may not fully capture oxidative progression, reinforcing the need for multi-marker strategies. Similarly, 2,4-bis(1,1-dimethylethyl)-phenol is a marker of quality deterioration in specialty coffees, including Ethiopian Yirgacheffe. Its accumulation is associated with plastic-like and phenolic off-odors, although concentration trends may decline after extended storage under controlled temperatures, indicating complex formation–degradation equilibria^10,39^.

More generally, storage leads to an increase in phenolic compounds such as 4-vinylguaiacol and 4-ethyl-2-methoxyphenol, probably formed from the oxidation of chlorogenic acids and lignin-related compounds. These transformations are amplified under accelerated conditions (e.g., 50 °C), intensifying earthy and smoky attributes. Concurrently, volatile sulfur compounds and heterocyclic aroma contributors including aldehydes, sulfides, alcohols, and furans undergo significant depletion through evaporation and oxidative conversion into less volatile species. The rapid loss or transformation of dimethyl sulfide exemplifies this phenomenon, contributing to attenuation of the characteristic roasted profile^12^.

Staling kinetics are strongly modulated by storage conditions and matrix configuration. Temperature exerts the most pronounced effect on sensory and chemical stability (*p* < 0.0001)^31^, with elevated temperatures (50–60 °C) markedly accelerating VOCs depletion and reducing aroma diversity. In contrast, refrigerated (4 °C), frozen ( − 20 °C), or controlled room-temperature storage better preserves compositional integrity^12^. Physical form further dictates oxidative exposure: ground coffee, characterized by increased surface area and oxygen permeability, exhibits substantially faster degradation than whole beans, frequently reaching unacceptable sensory scores within weeks under high-temperature conditions. Whole beans, by contrast, demonstrate comparatively greater resilience under equivalent storage regimes^31^.

Post-harvest processing also influences shelf stability by modifying the composition of aroma precursors. Dry-processed coffees may exhibit reduced phenolic escalation during storage, whereas wet-processed samples are more susceptible to ester hydrolysis, resulting in diminished fruity and floral notes. These matrix-dependent effects highlight the importance of integrating chemical monitoring with processing history^12^.

### Origin, authenticity via chemometrics and machine learning

Authentication of geographical origin and botanical species constitutes a strategic priority in coffee science, particularly for high-value specialty products where certified provenance can substantially increase market value^9^. In this context, HS–SPME–GC–MS is a leading volatilomic fingerprinting platform, providing a non-destructive and environmentally responsible approach to provenance verification^13^. It selectively extracts volatile secondary metabolites influenced by agro-ecological factors such as altitude, solar radiation, and rainfall, which allows the differentiation of terroir at the molecular level.

In green Arabica beans, volatile fingerprints reflect region-specific metabolic adaptations. Studies of Ethiopian coffees demonstrate that altitude and microclimate significantly modulate precursor accumulation; that is, high-altitude Amaro samples are characterized by elevated methyl salicylate, 1-hexanol, and nonanal, whereas lower-altitude Bebeka origins show higher levels of (+)-4-carene and cineole^6^. Comparable altitude-dependent differentiation has been reported in Brazilian Arabica, where variations in aldehydes and ketones contribute to distinctive aromatic signatures. These findings show that changes in precursor composition caused by environmental factors can be measured and used for analytical purposes^36^.

Although roasting amplifies chemical complexity through Maillard reactions and caramelization, the resulting VOCs remain constrained by the initial precursor composition of green beans^10,32^. Pyridine, for example, has been proposed as a discriminant marker for continental origin in roasted coffee, reportedly independent of roast degree. Similarly, in premium commodities such as *Luwak* (civet) coffee, HS–SPME–GC–MS profiling distinguishes bio-transformed beans from conventional Arabica through elevated phenolic derivatives and characteristic furan patterns. Nevertheless, single-marker interpretation should be approached cautiously, as processing variables and storage conditions may confound classification^37^.

**Fig. 4:**
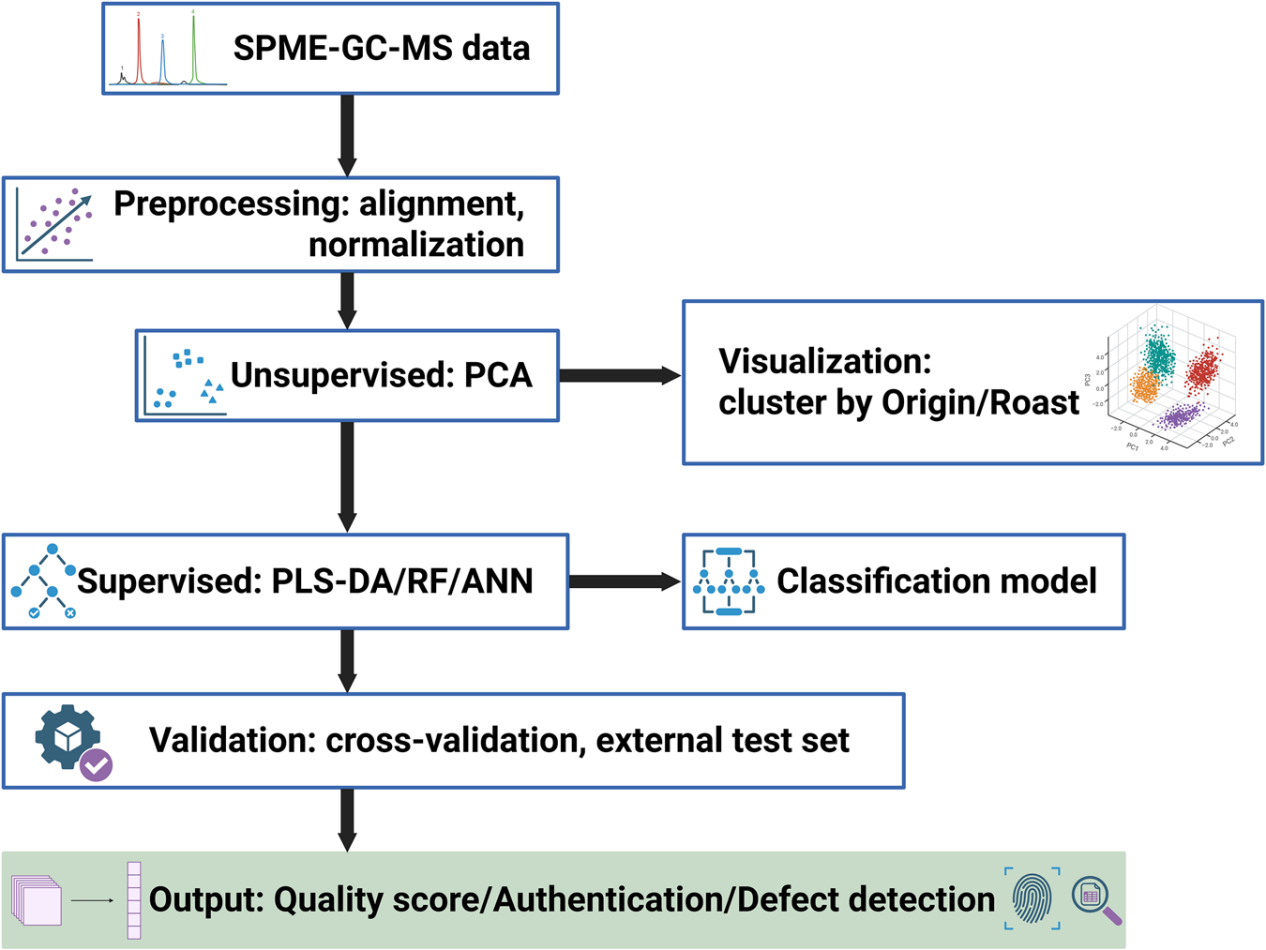
Chemometrics data analysis workflow for SPME-GC-MS coffee profiling. While traditional chemometric approaches, such as PCA and PLS-DA, provide reliable classification based on linear patterns within SPME–GC–MS fingerprint^32,37^, recent advances in machine learning further expand the analytical landscape. Algorithms such as random forest and artificial neural networks have demonstrated superior predictive accuracy in origin classification studies, including Indonesian coffees, by modeling nonlinear relationships and ensemble decision structures. Integration with portable technologies (e.g., NIR spectroscopy, electronic noses) has enabled fast in situ prediction of volatile composition with strong correlation coefficients (R > 0.95). However, model robustness remains contingent upon representative training datasets and standardized sampling protocols^15,38,39^.

As shown in Fig. 4, transition from descriptive profiling to robust authentication requires integration with chemometric modeling. Unsupervised methods such as principal component analysis (PCA) are routinely employed to visualize clustering and to relate volatile variables to botanical (Arabica, Robusta) and regional groupings^9,13^. However, PCA primarily shows variance structure rather than predictive capacity. Supervised approaches, including linear discriminant analysis and partial least squares discriminant analysis (PLS-DA), offer enhanced classification performance and enable identification of influential discriminant markers (e.g., acetic acid, furfural, 2-formylpyrrole)^1^. Reported high training-set accuracies, sometimes approaching 100%, demonstrate potential but also necessitate rigorous external validation to mitigate overfitting^13^.

## Green assessment

**Fig. 5:**
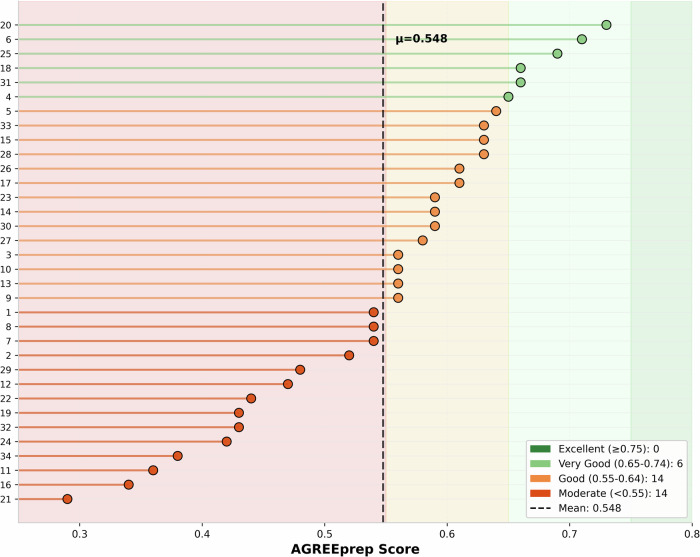
AGREEprep greenness scores for 34 SPME methods applied to coffee quality assessment, ranked by total score. The X-axis shows the distribution of scores across four greenness categories: Excellent (≥0.75), Very Good (0.65–0.74), Good (0.55–0.64), and Moderate (<0.55). The dashed vertical line indicates the mean score (μ = 0.548). The Y-axis represents the articles evaluated according to Table 1.

The greenness of sample preparation strategies employed in coffee volatile analysis was evaluated using the AGREEprep metric, a tool designed to assess the sustainability performance of analytical sample preparation procedures^27^. This metric evaluates ten criteria and operates on a penalty-point basis, where lower penalty points correspond to higher greenness scores, with final scores ranging from 0 (least green) to 1 (most green)^47^. The analysis of the SPME-based methods reported in recent coffee research showed a mean AGREEprep score of 0.548 (see Fig. 5 and **Supplementary Material**), with study scores ranging from 0.29 to 0.73 (see Table 1). This distribution indicates that SPME methodologies generally achieve moderate to high greenness performance, with substantial variability attributable to differences in experimental design, instrumentation, and operational parameters. The four greenness categories applied in this review follow the score ranges used in previous AGREEprep applications^48,49^, where scores closer to 1 correspond to greener procedures. The “excellent” category (≥0.75) corresponds to procedures whose penalty points sum to less than 0.25 across the ten criteria. The “very good” category (0.65–0.74) and the “good” category (0.55-0.64) include procedures with progressively higher penalty contributions, while the “moderate” category (<0.55) groups methods whose total penalty exceeds 0.45. Applied to the dataset of 34 SPME methods, no method reached the excellent threshold because the maximum observed score was 0.73. The remaining methods were distributed as follows: very good, 18% (6 of 34 methods); good, 41% (14 of 34); and moderate, 41% (14 of 34). The mean score of 0.55 lies at the boundary between the good and moderate categories, which is consistent with the observation that solvent-free extraction and fiber reusability raise the score, while energy use during desorption and the small but unavoidable use of conditioning solvents keep most methods below the very good threshold.

A principal advantage of SPME is the elimination of organic solvents during the extraction phase, representing a fundamental departure from conventional LLE and SPE methodologies that typically require substantial volumes of hazardous solvents such as dichloromethane, diethyl ether, or methanol^13,14^. In the evaluated coffee studies, Criterion 2 of AGREEprep (Use of safer reagents and solvents) demonstrated low penalty points, with 14 methods (47.01%) receiving zero penalty points and the remaining showing only minor deductions (mean penalty = 0.06) (see **Supplementary Material**). This pattern reflects the inherent solvent-free nature of HS–SPME, where analyte enrichment occurs through direct partitioning between the sample matrix and the polymeric fiber coating without liquid extraction media^32^.

The environmental and occupational health implications of solvent elimination are substantial. Traditional solvent-based extraction methods for coffee volatiles can consume 50–500 mL of organic solvent per sample, generating corresponding volumes of hazardous waste requiring specialized disposal^13,14^. The elimination of this solvent requirement reduces the environmental footprint of coffee quality analysis. At the same time, it minimizes analyst exposure to toxic vapors and decreases the need for fume hood infrastructure. This alignment with green analytical chemistry principles makes SPME a suitable sample preparation method for laboratories aiming to work more sustainably^29^. It is noteworthy that while the extraction phase is solvent-free, some studies employed minimal solvent volumes (typically <1 mL methanol) for internal standard preparation or fiber conditioning, resulting in minor penalty point assignments. However, these amounts represent a reduction of more than 99% compared to conventional extraction methods, showing the impact of SPME on reducing solvent use in coffee volatile analysis.

### Reduced waste and energy use

The environmental benefits of SPME go beyond eliminating solvents and include important reductions in waste and energy use. Analysis of Criterion 4 (Waste Production) across the evaluated studies revealed generally favorable performance, with mean penalty points of 0.08 (see **Supplementary Material**). Most methods (*n* = *27*, 79.4%) achieved penalty points ≤0.10, indicating minimal waste generation. The primary waste streams in SPME-based coffee analysis are limited to the sample matrix itself (typically 0.5–2.0 g of coffee), disposable vial septa, and occasional fiber replacement after extended use cycles.

Energy consumption, evaluated under Criterion 8, demonstrated mean penalty points of 0.03, with 26 methods (76.5%) receiving the minimum penalty of 0.02-0.03 points (see **Supplementary Material**). This good environmental profile is related to the moderate temperatures used in SPME extraction, which typically operates between 30–75 °C for 10-90 minutes^14,19,20^. Compared with thermal desorption or steam distillation methods that require high temperatures for long periods, SPME uses less energy. The reduction in both waste and energy use makes SPME a method that supports the United Nations Sustainable Development Goals (SDGs), especially SDG 12 (Responsible Consumption and Production) and SDG 13 (Climate Action)^50^. For high-throughput coffee quality control laboratories processing hundreds of samples daily, the cumulative environmental benefits of adopting SPME methodologies are considerable.

### Reusability of fibers

SPME fibers can be reused, which is an important sustainability advantage and meets Criterion 3 of the AGREEprep tool (sustainable, reusable, and renewable materials). Commercially available SPME fibers, particularly those coated with DVB/CAR/PDMS or PDMS/DVB, can withstand hundreds of thermal conditioning cycles without significant degradation in extraction performance^10,16^. Among the evaluated studies, most received zero or minimal penalty points (≤0.02) for this criterion (see **Supplementary Material**), reflecting the established reusability of fiber coatings.

The practical implications of fiber reusability are substantial from both economic and environmental perspectives. A single SPME fiber can replace thousands of disposable SPE cartridges or solvent extraction vessels over its operational lifetime^10^. This longevity translates to reduced material consumption, lower per-sample costs, and decreased waste generation. Studies have demonstrated that DVB/CAR/PDMS fibers maintain consistent extraction efficiency for coffee volatiles across more than 200 analytical cycles when proper conditioning protocols are followed^16^.

However, fiber durability is contingent upon appropriate handling and storage conditions. Exposure to aggressive matrices, mechanical damage, or improper thermal conditioning can compromise coating integrity and necessitate premature replacement. The development of SPME Arrow technology, with its enhanced mechanical stability and larger sorbent phase, represents an advancement that further extends fiber lifetime while simultaneously improving analytical sensitivity^5,15,21^.

### Simplified workflows

SPME simplifies the analytical workflow compared to conventional extraction methods, which helps achieve good AGREEprep scores in criteria related to procedural efficiency. Criterion 7 (Process Steps or Automation) showed mean penalty points of 0.05, with 24 methods (70.5%) achieving scores ≤0.05. This reflects the streamlined nature of SPME workflows, which involve sample weighing, brief equilibration, fiber exposure, and direct thermal desorption, eliminating the multiple extraction, concentration, and cleanup steps required by solvent-based methods^32^. The simplified workflow enhances sample throughput (Criterion 6), which demonstrated mean penalty points of 0.10 across evaluated studies. SPME methods typically achieve total analysis times of 30–90 min per sample, compared to 4–24 hours for conventional extraction approaches^13,15^. This efficiency is valuable for industrial quality control applications where rapid turnaround is essential for production decision-making. The compatibility of SPME with autosampler systems further enhances throughput by enabling unattended operation and overnight sample sequences^22^.

Post-sample preparation configuration (Criterion 9) showed favorable performance with mean penalty points of 0.05, reflecting the direct compatibility of SPME with standard GC–MS instrumentation without requiring additional concentration, derivatization, or cleanup steps. The thermal desorption process transfers extracted analytes directly to the chromatographic column, maintaining analytical sensitivity while minimizing sample manipulation^19,20^.

The integration of simplified SPME workflows with chemometric data analysis platforms creates opportunities for automated quality assessment systems capable of real-time classification and decision support^1,11,22,39,40^. This convergence of green sample preparation and intelligent data processing represents a paradigm shift toward sustainable, efficient, and intelligent coffee quality control systems suitable for Industry 4.0 manufacturing environments^22^.

## Critical analysis

### AGREEprep evaluation

Evaluation of greenness by AGREEprep gives a mean score of 0.548 across 34 methods. This result points to solvent-free extraction and fiber reuse as the main environmental strengths of SPME-based workflows, in agreement with the criterion-by-criterion analysis presented in Section 5. At the same time, the distribution of scores shows that no method reached the excellent category (≥0.75), which indicates that further improvements are still possible, particularly in energy consumption during desorption and in the small but non-negligible solvent volumes used for fiber conditioning and internal standard preparation. Wider use of complementary greenness metrics is still lacking, probably because current studies focus more on resolving complex volatile profiles than on full life-cycle assessment. Several limitations of the present synthesis should be acknowledged: the lack of standardized protocols reduces reproducibility across studies, the small datasets used in many machine learning applications increase the risk of overfitting, and the limited inclusion of Robusta and minor producing regions restricts broader application of the AGREEprep benchmark established here.

### Analytical and chemometric advances

**Fig. 6:**
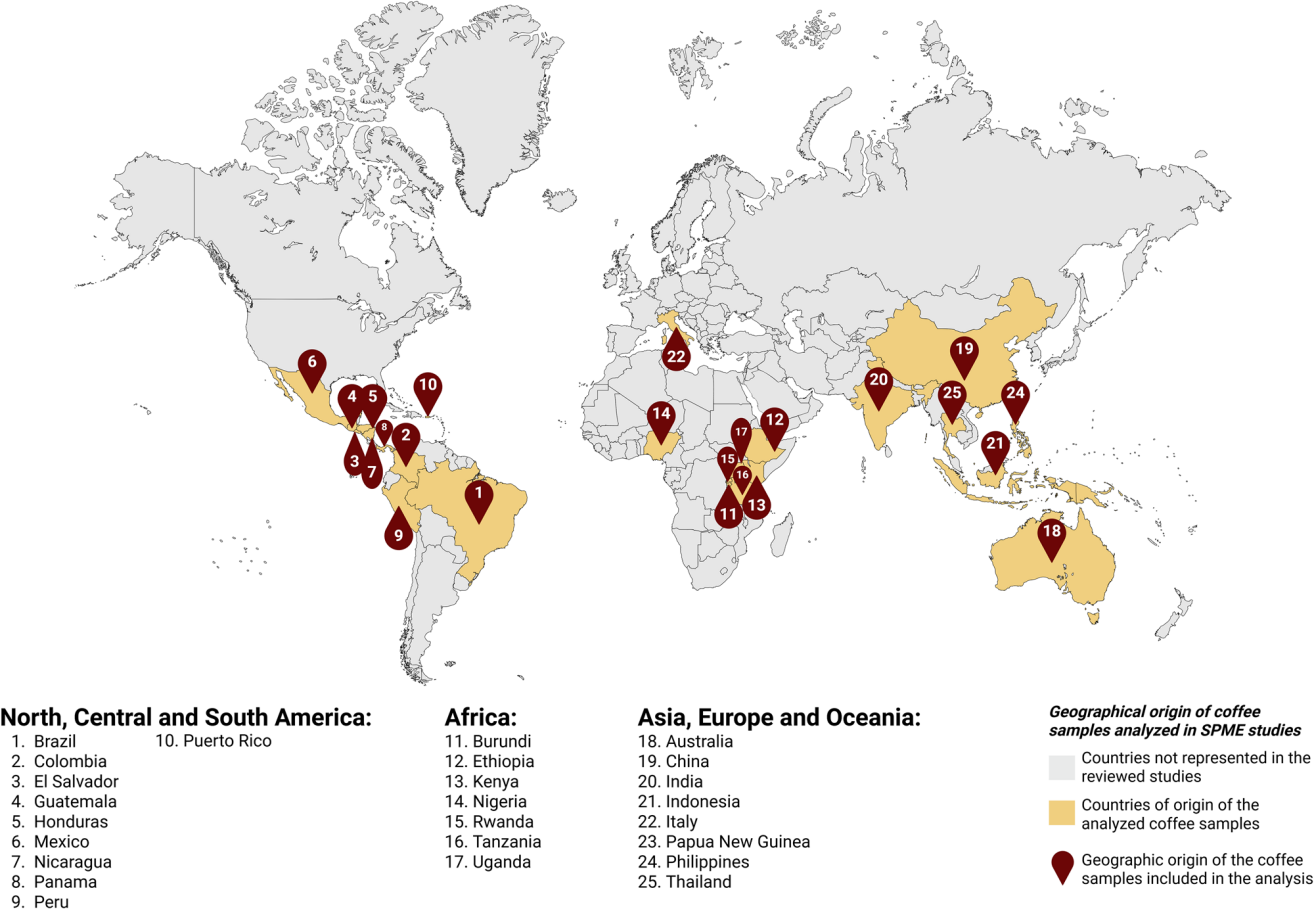
Geographic distribution of coffee-producing countries whose beans were used as study samples in SPME-based volatile analysis studies indexed in Scopus (2020–2025). Countries highlighted in yellow indicate origins from which coffee samples were sourced in at least one of the reviewed studies (see Table 1 for details); grey countries represent producing regions not covered by any study in the dataset. Pin markers indicate the specific country of sample provenance, numbered according to the regional lists below.

Over the past five years, coffee analysis has moved from mainly descriptive chemical studies to a data-driven analytical approach that combines untargeted VOC profiling with machine learning. In this context, machine learning models can predict sensory scores and geographical origin with accuracy above 90%, while SPME Arrow supports trace-level detection because its sorbent surface is five to twenty times larger than that of conventional fibers. As a result, HS-SPME-GC-MS has become the main analytical approach. Compared with LLE and SPE, this method avoids hazardous solvents, lowers solvent consumption by more than 99%, and operates at moderate temperatures of 30 to 75 °C with reusable fibers that can be used for hundreds of cycles. It therefore combines strong analytical performance with the principles of green analytical chemistry. The reviewed studies show several common trends. Most reports focus on furans and pyrazines as markers of roasting; they mainly study Arabica coffee from regions in North, Central, and South America, Europe, and Asia (see Fig. 6). They use ground roasted beans as the main sample type; they apply DVB/CAR/PDMS fibers for broad volatile extraction; and they direct the analysis toward origin authentication rather than purely descriptive chemical profiling. Similar strategies have also been applied to tea, wine, cocoa, and honey, where volatile composition is also complex and solvent-free extraction is preferred.

### From research methods to routine industrial practice

Despite the analytical advances summarized above, an important issue is the gap between advanced analytical methods and routine industrial practice. In commercial coffee production, quality control relies mainly on standardized sensory evaluation, especially the cupping protocol of the SCA, which guides grading and pricing. Physical tests for green coffee include moisture content, water activity, bean density, defect count, and color. Roasted coffee is evaluated by roast color, particle size distribution, gas content in packaging, dissolved solids, pH, and refractive index. Chemical testing mainly targets regulatory parameters such as ochratoxin A, pesticide residues, caffeine content, and microbiological safety, following AOAC and ISO methods. In the United States, the Food and Drug Administration regulates safety, while the Agricultural Marketing Service^51^ of the United States Department of Agriculture defines analytical requirements for procurement. By contrast, advanced methods such as HS-SPME-GC-MS combined with chemometric analysis are not widely used in routine industrial workflows. Industry favors faster and lower-cost techniques suitable for high-throughput testing. Adoption of SPME-based approaches at the industrial scale therefore requires standardized protocols, validated methods, inter-laboratory reproducibility, and clear cost-benefit evidence. Closing this gap is essential to translate the research advances summarized in “AGREEprep evaluation” and “Analytical and chemometric advances” into practical quality control systems while preserving the environmental advantages of green analytical methods.

## Conclusions and perspectives

SPME is a green sample preparation technique for coffee quality assessment, delivering substantial environmental benefits while maintaining analytical performance. AGREEprep evaluation of 34 methods demonstrated moderate to high greenness (mean score 0.55 ± 0.11), with exceptional performance in solvent elimination (47.01% of methods achieving zero penalty points), material reusability (91.2% with minimal penalties), and energy efficiency. The complete elimination of organic solvents during extraction reduces hazardous waste by >99% compared to conventional LLE and SPE approaches, while fiber reusability across hundreds of cycles minimizes consumable consumption and operational costs. Coffee serves as an exemplary benchmark matrix for validating green analytical methodologies, given its global economic significance, complex volatile profile (>1000 compounds), and multidimensional quality attributes spanning origin, processing, roasting, and sensory characteristics. The demonstrated capability of SPME to profile these diverse attributes coupled with its compatibility with chemometric modeling and portable technologies establishes a template for green quality control applicable across the food industry.

Over the next decade, coffee analysis may move from isolated laboratory studies to integrated industrial systems. In this setting, technological progress is expected to center on automation, with robotic SPME systems connected directly to roasting lines for real-time quality adjustment. However, this step still depends on solving current problems related to fiber durability in oily matrices. Another promising direction is vacuum-assisted SPME, in which reduced headspace pressure improves mass transfer. This approach has already been validated for complex food matrices such as fruits and fermented beverages, which suggests that it may also be useful for coffee by improving the recovery of trace odorants under lower thermal stress^52^. At the same time, sustainability evaluation should move beyond AGREEprep and include additional tools such as the blue applicability grade index (BAGI) and the red analytical procedure index (RAPI), to provide an overview of the practicality and performance, respectively^53,54^. The use of these multi-dimensional frameworks in coffee studies would give a more complete environmental profile and help identify specific problem areas, such as high energy demand at elevated temperatures or hazardous reagent exposure during derivatization. In parallel, miniaturization is another major direction, with portable GC–MS instruments and smartphone-based electronic noses offering new possibilities for quality control at the site of origin. Despite this progress, major barriers still limit industrial use, because most reviewed studies present proof-of-concept methods based on small and homogeneous sample sets rather than protocols suited to high-throughput commercial roasting. Closing this gap requires proof that these methods remain reliable under real processing conditions, together with lower cost per analysis. Moreover, the field needs harmonized international standards for fiber specifications and multi-metric greenness reporting so that analytical methods give consistent results across global industrial settings and support objective quality assessment throughout the commercial coffee supply chain.

## Supplementary information

Supplementary material.

## Data Availability

No datasets were generated or analyzed during the current study.
